# Fidgety Movement Classification in Preterm Infants Without Major Structural Brain Abnormalities: Associations with Gestational Age, Birth Weight, and Neonatal Morbidity

**DOI:** 10.3390/children13091259

**Published:** 2026-09-16

**Authors:** Bruna Bašić, Ana Katušić, Ruža Grizelj, Ana-Marija Bohaček

**Affiliations:** 1 Early Motor Behaviour Research Laboratory, Faculty of Education and Rehabilitation Sciences, University of Zagreb, 10000 Zagreb, Croatia; bruna.basic@erf.unizg.hr (B.B.); ana-marija.bohacek@erf.unizg.hr (A.-M.B.); 2 School of Medicine, University of Zagreb, 10000 Zagreb, Croatia; ruza.grizelj@kbc-zagreb.hr; 3 Department of Pediatrics, University Hospital Centre Zagreb, 10000 Zagreb, Croatia

**Keywords:** preterm infants, General Movements Assessment, fidgety movements, biological maturity, neonatal morbidity

## Abstract

**Highlights:**

**What are the main findings?**

- Infants with absent fidgety movements had lower gestational age and birth weight and longer respiratory treatment than those with abnormal fidgety movements.
- Infants with abnormal fidgety movements descriptively had the highest gestational age and birth weight and the shortest respiratory treatment among the three groups.

**What are the implications of the main findings?**

- Abnormal and absent fidgety movements should be analysed separately.
- Clinical factors do not indicate an ordinal risk gradient across fidgety movement classifications.

**Abstract:**

**Background/Objectives**: Fidgety movements are established markers of early neurological function in preterm infants. However, associated biological and neonatal factors remain insufficiently understood, particularly in infants without major structural brain abnormalities. This study aimed to compare biological maturity and neonatal morbidity in infants with normal, abnormal, and absent fidgety movements. **Methods**: Prospectively collected data from 57 preterm infants assessed at 12–16 weeks corrected age using Prechtl’s General Movements Assessment were analysed. Infants with moderate or severe abnormalities on term-equivalent magnetic resonance imaging were excluded. Gestational age, birth weight, clinically significant morbidities, a cumulative morbidity count, and durations of non-invasive respiratory support and mechanical ventilation were compared by fidgety movement classification. **Results**: Significant overall differences were found for gestational age (*p* = 0.018), birth weight (*p* = 0.027), non-invasive respiratory support (*p* = 0.014), and mechanical ventilation (*p* = 0.016). Infants with absent fidgety movements had lower gestational age (mean difference, 2.6 weeks; adjusted *p* = 0.021), lower birth weight (mean difference, 386 g; adjusted *p* = 0.021), longer non-invasive respiratory support (adjusted *p* = 0.014), and longer mechanical ventilation (adjusted *p* = 0.019) than infants with abnormal fidgety movements. Comparisons involving infants with normal fidgety movements were not significant. Individual morbidities and the cumulative morbidity count also did not differ by classification. **Conclusions**: Biological maturity and respiratory-treatment duration differed according to fidgety movement classification. However, the observed pattern did not indicate a simple ordinal relationship between the examined clinical characteristics and normal, abnormal, and absent fidgety movements.

## 1. Introduction

Spontaneous motor behaviour provides an early window into the functioning of the developing nervous system. According to the Neuronal Group Selection Theory, infants initially produce a broad repertoire of spontaneous movements and gradually select patterns that are better adapted to their internal state and environment [1,2]. The variability, complexity, and fluency of these movements reflect the infant’s ability to generate and modulate motor activity and may reveal disturbances in neural organization before overt neurological signs emerge [3]. The General Movements Assessment (GMA) evaluates these age-specific whole-body movement patterns and is a well-validated method for assessing early neurological function in term and preterm infants [3,4].

Fidgety movements are a distinct form of general movements (GMs); they typically occur between 9 and 20 weeks post-term age. Normal fidgety movements are continual movements of small amplitude and moderate speed, with variable acceleration and direction, involving the neck, trunk, and limbs. Abnormal fidgety movements retain the fidgety character but appear exaggerated in amplitude, speed, or jerkiness, whereas absent fidgety movements indicate that this age-specific pattern is not observed [3,5]. The absence of fidgety movements is one of the strongest early predictors of cerebral palsy [6,7] and has also been associated with later cognitive and broader neurodevelopmental difficulties [8,9]. Abnormal fidgety movements may also have developmental relevance, but their prognostic meaning appears less specific [5]. Therefore, abnormal and absent fidgety movements should not necessarily be treated as equivalent manifestations of a single non-normal category. Distinguishing between these patterns may be clinically relevant, as differences in their prognostic implications can inform developmental surveillance, communication with families, and decisions regarding referral for further assessment and early intervention [5,7].

Preterm birth interrupts a period of rapid brain development and increases the risk of motor, cognitive, sensory, and behavioural difficulties throughout childhood [10,11,12,13]. This risk generally increases as gestational age (GA) decreases [14], although adverse developmental outcomes may occur even among infants without major structural brain injury on term-equivalent magnetic resonance imaging (MRI) [15,16]. During the final trimester of gestation, the brain undergoes rapid cortical expansion, synaptogenesis, myelination, and maturation of thalamocortical and corticospinal pathways [15,17]. Following preterm birth, these processes continue in the extrauterine environment during a period of heightened vulnerability [18]. Because spontaneous movements arise from activity within brainstem and spinal networks under supraspinal modulation, their quality may be sensitive to the maturity and organization of the developing nervous system [2,19]. GA and birth weight (BW) may therefore provide clinically accessible, although partly overlapping, information about maturity and fetal growth that is relevant to fidgety movement quality.

Preterm infants also experience neonatal complications and intensive care interventions that may affect cerebral maturation. Bronchopulmonary dysplasia (BPD), prolonged respiratory support, neonatal sepsis, necrotizing enterocolitis (NEC), and retinopathy of prematurity (ROP) have been associated with adverse neurodevelopmental outcomes [20,21,22]. These associations may involve mechanisms related to systemic inflammation, oxidative stress, intermittent hypoxia, and disrupted neural maturation [15,23,24]. These conditions often co-occur and are more common at lower GA [14,20,25], making it difficult to separate their contributions from those of biological immaturity. However, the type, severity, and duration of neonatal exposures may help explain why infants born at a similar GA display different patterns of spontaneous motor behaviour. Measures of exposure duration, such as days of respiratory support or mechanical ventilation, may also provide information that is not captured by the presence or absence of a neonatal diagnosis.

Available evidence on the biological and neonatal factors associated with GM quality remains limited and difficult to compare. In a large and clinically heterogeneous cohort, Ma et al. [26] found that lower GA, lower BW, and severe birth asphyxia were independently associated with non-normal GMs during the fidgety period. However, the broad range of GA and clinical risk profiles limited conclusions about preterm infants without major structural brain abnormalities. In a cohort of very preterm infants, Peyton et al. [27] similarly reported lower GA and BW among infants with aberrant compared with normal fidgety movements. Their aberrant category, however, combined sporadic, abnormal, and absent fidgety movements, limiting direct comparison with the three-category approach used in the present study. Merino-Andrés et al. [28] examined GMs during the writhing period in moderate-to-late preterm infants. Composite neonatal risk scores were not associated with GM patterns, but longer respiratory support increased the likelihood of cramped-synchronized movements. This result suggests that the duration of a specific neonatal exposure may be more informative than an aggregate risk score. At fidgety age, Domagalska-Szopa et al. [29] found that absent fidgety movements in preterm infants were associated with severe neonatal conditions, including birth asphyxia, BPD, periventricular leukomalacia, and severe intraventricular haemorrhage. Abnormal fidgety movements were associated with a broader combination of neonatal, prenatal, and maternal factors. Their high-risk cohort included infants with severe cerebral lesions, which limits the applicability of these findings to infants without moderate or severe structural brain abnormalities.

Taken together, previous studies suggest possible links between biological maturity, neonatal exposures, and GM quality, although findings across studies have varied. Fjørtoft et al. [30] reported no association between motor repertoire and either GA or BW in infants born extremely preterm and/or with extremely low BW after excluding those with severe abnormalities on neonatal ultrasound. Differences in findings across studies may partly reflect variation in GA range, timing and approach to GM assessment, definitions of neonatal risk, and inclusion of infants with severe cerebral lesions [26,27,28,29,30]. Moreover, some studies have combined abnormal and absent fidgety movements, despite their potentially different clinical and prognostic implications. It therefore remains unclear whether preterm infants without moderate or severe structural brain abnormalities differ in biological maturity and neonatal morbidity according to fidgety movement classification. Examining normal, abnormal, and absent fidgety movements separately may clarify whether these categories are associated with different clinical characteristics in infants without major structural brain injury.

The present study therefore aimed to compare GA, BW, and indicators of neonatal morbidity among preterm infants with normal, abnormal, and absent fidgety movements, excluding infants with moderate or severe structural brain abnormalities. We hypothesized that infants with absent or abnormal fidgety movements would have a lower GA and BW and greater neonatal morbidity than infants with normal fidgety movements.

## 2. Materials and Methods

### 2.1. Study Design and Setting

This observational study represents a secondary analysis of prospectively collected data from an ongoing single-centre cohort of preterm infants enrolled in a standardized neurodevelopmental follow-up programme after discharge from the neonatal intensive care unit (NICU) at the University Hospital Centre Zagreb, Croatia. Infants included in the present analysis were recruited between September 2024 and May 2026. Neurodevelopmental assessments were conducted at the Early Motor Behaviour Research Laboratory, Faculty of Education and Rehabilitation Sciences, University of Zagreb.

### 2.2. Participants

Infants born before 37 completed weeks of gestation were consecutively recruited through the institutional neurodevelopmental follow-up programme. Infants were eligible for the analysis if they had (i) a gestational age at birth of <37 completed weeks; (ii) brain MRI performed at term-equivalent age; (iii) an assessable GMA recording obtained during the fidgety movement period, between 12 and 16 weeks corrected age (CA); and (iv) written informed consent provided by a parent or legal guardian.

Infants were excluded if they had congenital malformations, chromosomal or genetic abnormalities, severe sensory impairments, severe fetal growth restriction (FGR), or moderate or severe structural brain abnormalities on term-equivalent MRI.

MRI findings were evaluated using the Kidokoro scoring system [31], which provides a standardized assessment of abnormalities involving the cerebral white matter, cortical grey matter, deep grey matter, and cerebellum. A global brain abnormality score was calculated and classified as normal (0–3), mildly abnormal (4–7), moderately abnormal (8–11), or severely abnormal (≥12). Images were independently evaluated by experienced neuroradiologists who were blinded to the infants’ GMA findings and neonatal clinical data. Infants with moderate or severe MRI abnormalities were excluded from the present analysis.

**Figure 1:**
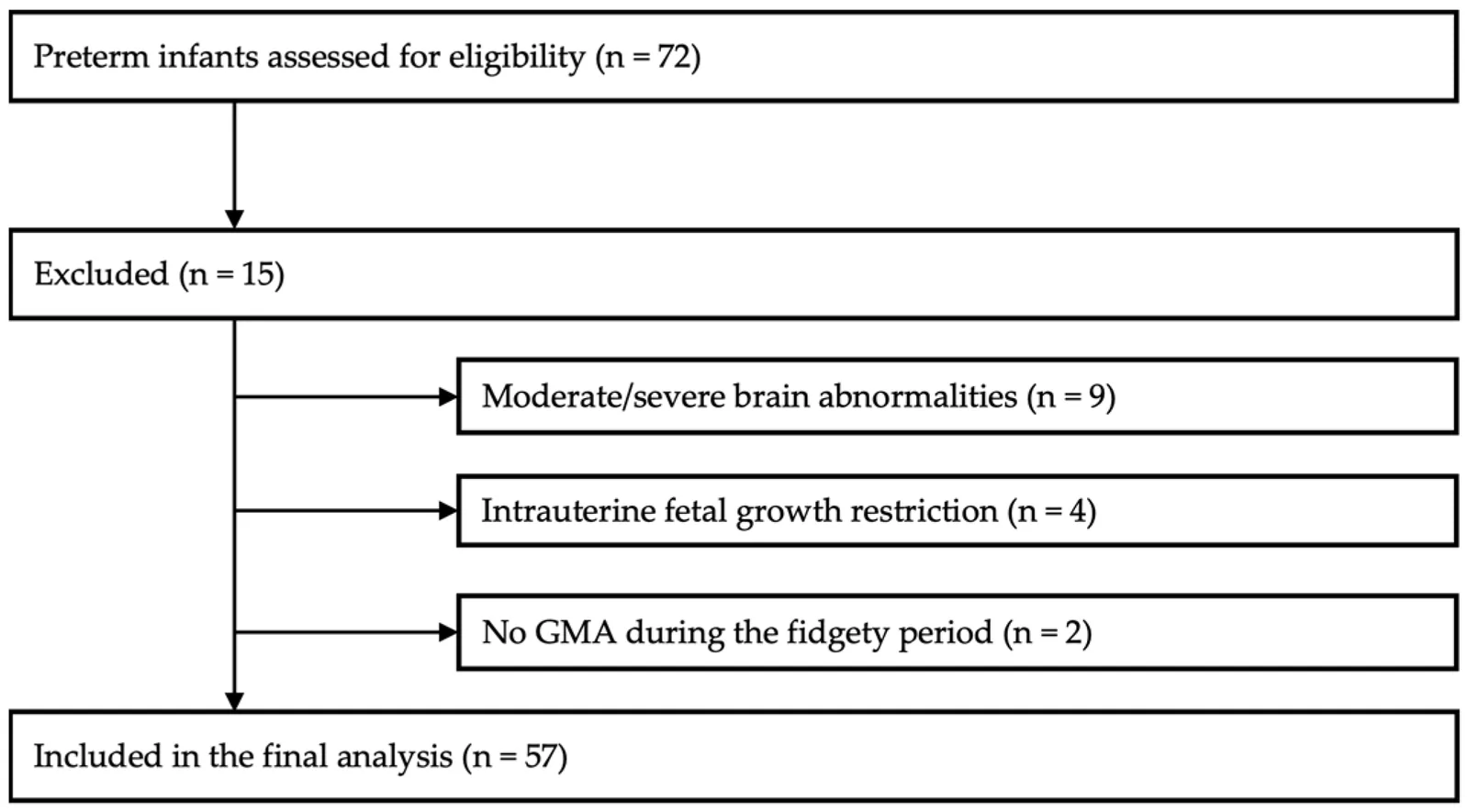
Flow diagram of participant selection and inclusion in the final analysis.

A total of 57 preterm infants met the eligibility criteria and were included in the final analysis. The participant selection process and reasons for exclusion are presented in Figure 1.

### 2.3. General Movements Assessment

Spontaneous motor behaviour was assessed using Prechtl’s General Movements Assessment (GMA) at 12–16 weeks CA, according to Prechtl’s standardized methodology. Infants were video-recorded in the supine position during active wakefulness, wearing minimal clothing and without external stimulation, handling, pacifiers, or interaction with the caregiver or examiner. Each recording lasted approximately 3 min. Recordings were repeated when the infant was crying, persistently drowsy, or insufficiently observable.

Video recordings were independently rated by two certified GMA assessors with advanced training. Assessors were blinded to neonatal and MRI data.

Fidgety movements were classified according to established criteria as normal, abnormal, or absent [3]. Initial interrater agreement was assessed using Cohen’s κ. The agreement between the two assessors was almost perfect (κ = 0.94). When initial classifications differed, the final classification was reached by consensus.

### 2.4. Clinical Variables

Perinatal and neonatal data were prospectively collected from hospital records as part of the cohort protocol.

Markers of biological maturity included GA at birth and BW. Gestational age, recorded in completed weeks and days, was converted into decimal weeks for statistical analyses, and BW was recorded in grams.

Neonatal morbidities included BPD, NEC, neonatal sepsis, and ROP. Each morbidity was described according to its recorded clinical severity. For comparisons of clinically significant neonatal morbidities across fidgety movement groups and for calculation of the cumulative morbidity count, predefined severity thresholds were applied as described below.

BPD was defined according to the National Institute of Child Health and Human Development consensus definition proposed by Jobe and Bancalari [32]. BPD required supplemental oxygen at a concentration greater than 21% for at least 28 days. For infants born before 32 weeks’ gestation, severity was determined at 36 weeks’ postmenstrual age or at discharge, whichever occurred first. For infants born at or after 32 weeks’ gestation, severity was determined at 56 days of postnatal age or at discharge, whichever occurred first. BPD was classified as mild when the infant was breathing room air at the time of assessment, moderate when supplemental oxygen below 30% was required, and severe when supplemental oxygen of at least 30% and/or positive-pressure respiratory support was required [32]. All BPD grades were included in the descriptive presentation of the cohort. However, only moderate or severe BPD was classified as clinically significant for group comparisons and for the cumulative neonatal morbidity count.

NEC was classified according to the modified Bell staging criteria [33]. Bell stage I was considered suspected rather than confirmed NEC and was therefore coded as absence of NEC. Only Bell stage II or III was classified as confirmed NEC and included in the analyses of clinically significant morbidity [33].

Confirmed neonatal sepsis was defined as clinical signs consistent with systemic infection together with isolation of a pathogenic microorganism from a blood culture [34]. Clinically suspected or culture-negative episodes were not classified as neonatal sepsis.

ROP was classified according to the recorded ophthalmological findings. Any-stage ROP was included in the descriptive presentation of the cohort. Treatment-requiring ROP was defined as ROP that required intravitreal anti-vascular endothelial growth factor treatment [35] and was classified as clinically significant for group comparisons and for the cumulative neonatal morbidity count. Mild ROP that did not require treatment was not included in the clinically significant morbidity category.

To describe the co-occurrence of clinically relevant neonatal complications, a cumulative neonatal morbidity count was calculated by summing four binary indicators: moderate-to-severe BPD, confirmed neonatal sepsis, NEC Bell stage II or III, and treatment-requiring ROP. Each component was coded as absent (0) or present (1), yielding a possible score from 0 to 4. Higher values indicated a greater number of co-occurring clinically relevant morbidities. The same weight was assigned to each component. This variable was therefore interpreted as an unweighted morbidity count rather than as a validated neonatal risk or illness-severity score.

The duration of non-invasive respiratory support was defined as the total number of days during which the infant received continuous positive airway pressure, non-invasive positive-pressure ventilation, or high-flow nasal cannula support. The duration of invasive mechanical ventilation was defined as the total number of days of conventional or high-frequency mechanical ventilation delivered through an endotracheal tube. Duration was initially recorded in hours and converted to days by dividing the total number of hours by 24.

### 2.5. Statistical Analysis

Data were analyzed using IBM SPSS Statistics (Version 27; IBM Corp., Armonk, NY, USA) [36].

Continuous variables were summarized using means and standard deviations when approximately normally distributed and medians and interquartile ranges when distributions were non-normal. Categorical variables were summarized using frequencies and percentages. Distributional assumptions were evaluated using Shapiro–Wilk tests and visual inspection of histograms and Q–Q plots. Homogeneity of variance was assessed using Levene’s test.

Differences among infants with normal, abnormal, and absent fidgety movements were examined separately for each biological and neonatal variable. Continuous variables meeting parametric assumptions and the assumption of homogeneity of variance were compared using one-way analysis of variance (ANOVA). When the assumption of homogeneity of variance was not met, Welch’s ANOVA was used. Non-normally distributed continuous variables and ordinal count variables were compared using the Kruskal–Wallis test. Categorical variables, including individual neonatal morbidities, were compared using Pearson’s χ^2^ test or the Fisher–Freeman–Halton exact test when expected cell frequencies were insufficient for Pearson’s χ^2^ test. For sparse contingency tables, exact *p*-values were estimated using a Monte Carlo procedure with 300,000 samples.

When an overall one-way ANOVA was statistically significant, pairwise group comparisons were performed using Tukey’s honestly significant difference test. When an overall Kruskal–Wallis test was statistically significant, pairwise comparisons were performed using Dunn’s post hoc test with Holm adjustment for multiple comparisons. Post hoc comparisons were not performed following non-significant omnibus tests.

Effect sizes were reported as eta squared (η^2^) for one-way ANOVA, epsilon squared (ε^2^) for Kruskal–Wallis tests, and Cramér’s V for categorical comparisons. Pairwise effect sizes were reported as Hedges’ g for parametric comparisons and r for Dunn’s comparisons. Effect size r was calculated as Z/√N, where N represented the combined number of infants in the two groups included in the corresponding pairwise comparison. Absolute r values < 0.10 were considered negligible, 0.10–0.29 small, 0.30–0.49 moderate, and ≥0.50 large. Hedges’ g values of approximately 0.20, 0.50, and 0.80 were interpreted as small, moderate, and large, respectively. All tests were two-sided, and statistical significance was set at *p* < 0.05.

## 3. Results

### 3.1. Sample Characteristics

The final study sample comprised 57 preterm infants. Participant flow is presented in Figure 1. Thirty-three infants (57.9%) were male. Median GA at birth was 29.1 weeks (IQR 27.4–32.0), and mean BW was 1389 ± 396 g. The cohort predominantly comprised very and extremely preterm infants, while overall, 40 infants (70.2%) were born before 32 weeks’ gestation.

Fifteen infants (26.3%) had BPD, including 10 (17.5%) with mild and five (8.8%) with moderate BPD. Four infants (7.0%) had NEC, all classified as Bell stage III. Four infants (7.0%) had treatment-requiring ROP. Additional perinatal and neonatal characteristics of the study sample are presented in Table 1.

GMA was performed at a median corrected age of 13.0 weeks (IQR 12.1–14.3). Fidgety movements were classified as normal in 24 infants (42.1%), abnormal in 11 (19.3%), and absent in 22 (38.6%). Term-equivalent MRI findings according to fidgety movement classification are presented in Table 2. The distribution of normal and mildly abnormal MRI findings did not differ significantly across the three groups, χ^2^(2) = 3.21, *p* = 0.201, Cramér’s V = 0.237.

Among the 28 infants with a mildly abnormal global MRI score, 20 (71.4%) had a non-zero cerebral white matter subscore. The most common white matter findings were mild lateral ventricular dilatation, thinning of the corpus callosum, and delayed myelination characterized by minimal myelination of the posterior limb of the internal capsule (PLIC).

Twelve infants (42.9%) had a non-zero deep grey matter subscore, reflecting focal signal abnormalities, while five (17.9%) had a non-zero cerebellar subscore attributable to punctate haemorrhagic signal abnormalities. No cortical grey matter abnormalities were identified. Some infants had abnormalities in more than one domain; therefore, these categories were not mutually exclusive.

### 3.2. Biological Maturity

**Figure 2:**
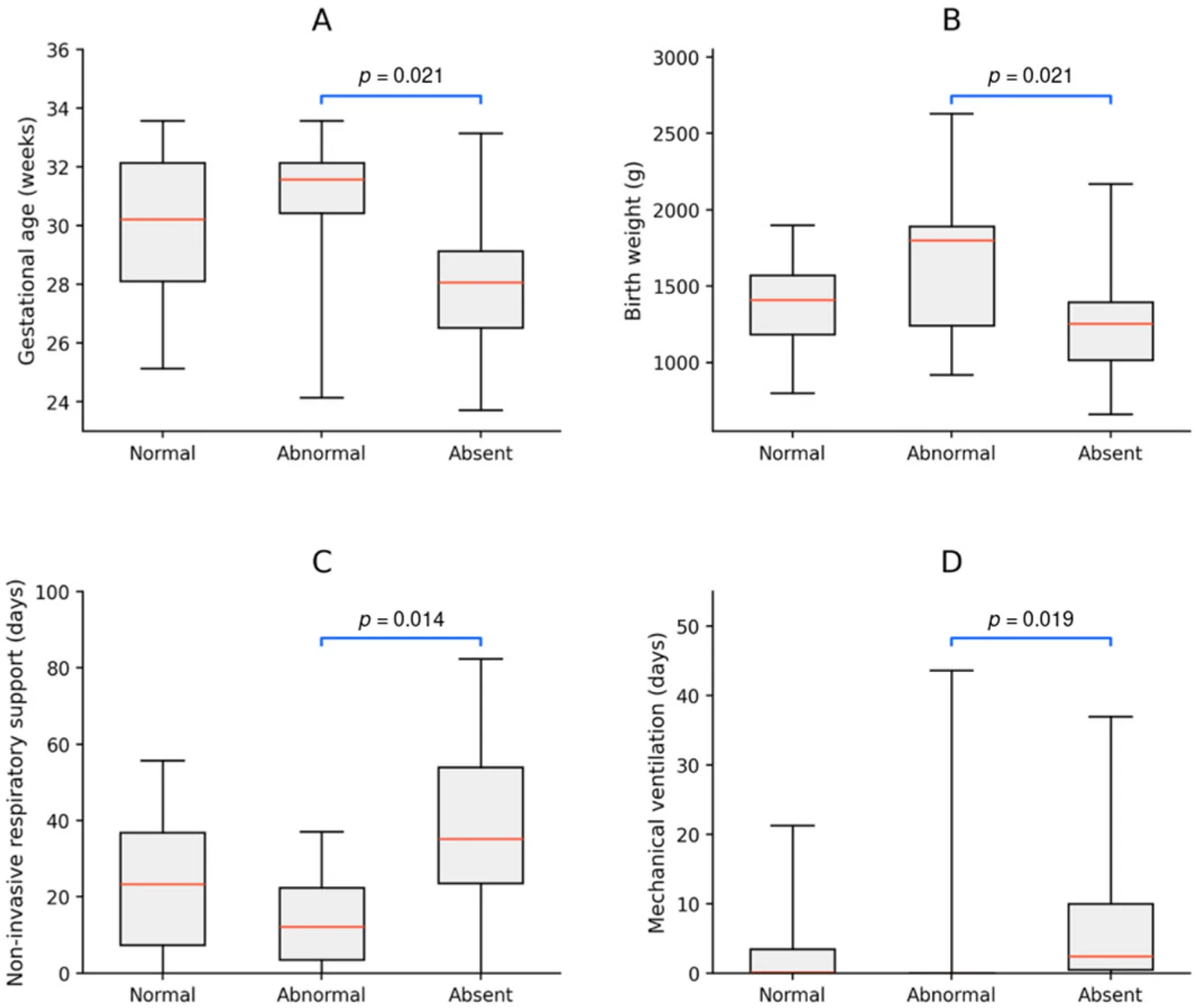
Distribution of gestational age (**A**), birth weight (**B**), duration of non-invasive respiratory support (**C**), and duration of mechanical ventilation (**D**) according to fidgety movement classification. Boxes represent the interquartile range, horizontal lines indicate the median, and whiskers represent the range of observed values. Brackets indicate statistically significant pairwise differences between infants with abnormal and absent fidgety movements. Pairwise comparisons for gestational age and birth weight were based on Tukey’s post hoc tests. Meanwhile, comparisons for respiratory support and mechanical ventilation were based on Dunn’s post hoc tests with Holm adjustment. Displayed *p*-values are multiplicity-adjusted.

Gestational age differed significantly among infants with normal, abnormal, and absent fidgety movements, F(2,54) = 4.35, *p* = 0.018, η^2^ = 0.139 (Table 3; Figure 2A). Mean GA was 29.9 ± 2.5 weeks in infants with normal fidgety movements, 30.9 ± 2.6 weeks in those with abnormal fidgety movements, and 28.3 ± 2.6 weeks in those with absent fidgety movements.

Tukey-adjusted post hoc comparisons showed that infants with absent fidgety movements were born at a significantly lower GA than infants with abnormal fidgety movements (mean difference = 2.6 weeks, adjusted *p* = 0.021, Hedges’ g = 0.979). Neither the normal–abnormal comparison nor the normal–absent comparison reached statistical significance after adjustment for multiple comparisons (Table 4).

Birth weight also differed significantly among the three fidgety movement groups, F(2,54) = 3.87, *p* = 0.027, η^2^ = 0.125 (Table 3; Figure 2B). Mean BW was 1375 ± 303 g in infants with normal fidgety movements, 1656 ± 502 g in those with abnormal fidgety movements, and 1270 ± 381 g in those with absent fidgety movements.

Tukey-adjusted comparisons showed that infants with absent fidgety movements had significantly lower BW than infants with abnormal fidgety movements (mean difference = 386 g, adjusted *p* = 0.021, Hedges’ g = 0.888). Birth weight did not differ significantly between infants with normal and abnormal fidgety movements (adjusted *p* = 0.110) or between those with normal and absent fidgety movements (adjusted *p* = 0.619) (Table 4).

### 3.3. Neonatal Morbidity

The duration of non-invasive respiratory support differed significantly across the three fidgety movement groups, H(2) = 8.52, *p* = 0.014, ε2 = 0.12 (Table 3, Figure 2C). Median duration was 23.3 days (IQR 7.4–36.9) in infants with normal fidgety movements, 12.2 days (IQR 3.5–22.4) in infants with abnormal fidgety movements, and 35.2 days (IQR 23.6–54.0) in infants with absent fidgety movements.

Dunn–Holm post hoc comparisons showed that infants with absent fidgety movements received non-invasive respiratory support for significantly longer than infants with abnormal fidgety movements (Holm-adjusted *p* = 0.014, r = 0.491) (Table 5). Neither the normal–abnormal comparison nor the normal–absent comparison reached statistical significance after adjustment for multiple comparisons.

The duration of invasive mechanical ventilation also differed significantly among the three fidgety movement groups, H(2) = 8.25, *p* = 0.016, ε2 = 0.116 (Table 3, Figure 2D). Median duration was 0.1 days (IQR 0.0–3.5) in infants with normal fidgety movements, 0.0 days (IQR 0.00–0.8) in infants with abnormal fidgety movements, and 2.5 days (IQR 0.5–9.9) in infants with absent fidgety movements.

Dunn–Holm post hoc comparisons showed that infants with absent fidgety movements required significantly more days of mechanical ventilation than infants with abnormal fidgety movements (Holm-adjusted *p* = 0.019, r = 0.363) (Table 5). Neither the normal–abnormal comparison nor the normal–absent comparison reached statistical significance.

The frequencies of individual clinically relevant neonatal morbidities did not differ significantly across the fidgety movement groups. None of the omnibus comparisons reached statistical significance for moderate BPD (*p* = 0.505), neonatal sepsis (*p* = 0.535), NEC Bell stage III (*p* = 0.595), or treatment-requiring ROP (*p* = 0.403) (Table 3).

The clinically relevant morbidity count also did not differ significantly across the fidgety movement groups, H(2) = 2.25, *p* = 0.324, ε^2^ = 0.005 (Table 3). The median count was 1.0 (IQR 0.0–1.0) in infants with normal fidgety movements, 0.0 (IQR 0.0–0.5) in infants with abnormal fidgety movements, and 1.0 (IQR 0.0–1.0) in infants with absent fidgety movements.

## 4. Discussion

This study compared biological maturity and neonatal morbidity among preterm infants with normal, abnormal, and absent fidgety movements, all of whom had normal or mildly abnormal findings on term-equivalent MRI. GA, BW, and the durations of respiratory support and mechanical ventilation differed significantly across the three fidgety movement groups. However, all significant pairwise differences occurred between infants with abnormal and absent fidgety movements. Compared with infants with abnormal fidgety movements, those with absent fidgety movements had a lower GA and BW and required longer non-invasive respiratory support and invasive mechanical ventilation. Infants with normal fidgety movements did not differ significantly from either of the other groups. Individual neonatal morbidities and the cumulative neonatal morbidity count also did not differ significantly across the groups.

Thus, the findings did not support the hypothesized pattern of lower biological maturity and greater neonatal morbidity among infants with absent or abnormal fidgety movements relative to those with normal fidgety movements. More broadly, the observed pattern did not follow a simple clinical severity gradient from normal to abnormal to absent fidgety movements.

GA differed significantly across the fidgety movement groups. Previous studies have reported lower GA to less favourable GM trajectories or absent fidgety movements [8,29]. Ma et al. also found an overall difference in GA across normal, abnormal, and absent fidgety movement groups, with the lowest mean GA observed among infants with absent fidgety movements [26]. However, their adjusted analysis combined abnormal and absent fidgety movements into a single non-normal outcome, preventing separate conclusions about these two movement patterns. Our findings are only partially consistent with available evidence. Infants with absent fidgety movements had a lower GA than those with abnormal fidgety movements, but neither group differed significantly from infants with normal fidgety movements.

BW also differed significantly across the three groups. Infants with absent fidgety movements had a significantly lower BW than those with abnormal fidgety movements, whereas neither group differed significantly from infants with normal fidgety movements. Ma et al. likewise reported an overall difference in BW across the three fidgety movement groups, with the lowest mean BW among infants with absent fidgety movements [26]. Other studies have also associated lower BW with less favourable general movement trajectories [29,37]. Although our findings are consistent with these reports regarding infants with absent fidgety movements, BW did not show a graded relationship across the three classifications.

GA and BW should be interpreted together as two related, clinically accessible markers of biological maturity, but they capture only selected aspects of this multidimensional construct. GA primarily reflects the duration of intrauterine development, whereas BW reflects both maturity and fetal growth. Infants born at the same GA may differ considerably in BW and growth status [38]. Biological maturity also encompasses the maturation of individual organ systems, including structural and functional brain maturation, which was not comprehensively assessed in the present study. The parallel differences in GA and BW between the abnormal and absent fidgety movement groups may therefore reflect related aspects of biological maturity rather than separate influences on fidgety movement quality. Because the analyses were unadjusted, we could not determine whether BW was associated with fidgety movement classification independently of GA. Accordingly, the absence of significant differences in GA and BW should not be interpreted as evidence that the groups were equivalent in overall biological maturity.

Taken together, the GA and BW findings do not support a straightforward progression of biological risk across normal, abnormal, and absent fidgety movements. One possible interpretation is that abnormal and absent fidgety movements may occur in infants with partly different clinical characteristics rather than representing successive levels of severity. The present analyses, however, cannot establish distinct clinical profiles. Domagalska-Szopa et al. [29] reported different patterns of clinical risk, with absent fidgety movements more often associated with severe postnatal factors and abnormal fidgety movements associated with a broader combination of prenatal, maternal, and neonatal factors. However, their high-risk cohort included infants with severe cerebral lesions, which limits direct comparison with our sample. Longitudinal evidence also suggests that abnormal fidgety movements may have neurodevelopmental relevance, although their prognostic implications appear less specific than those of absent fidgety movements [39]. Nevertheless, lower GA marks greater neurodevelopmental vulnerability and may influence the organization of spontaneous motor behaviour [18,40]. Lower GA has also been associated with less favourable gross motor development throughout infancy [41].

Differences were also observed in the respiratory course. Infants with absent fidgety movements required respiratory support and mechanical ventilation for longer than infants with abnormal fidgety movements. Comparisons involving infants with normal fidgety movements were not statistically significant. Merino-Andrés et al. reported that longer respiratory support was associated with a greater likelihood of cramped-synchronized general movements during the writhing period [28]. Other studies have linked severe BPD and broader neonatal morbidity with less favourable neonatal GM trajectories [37]. These studies differ from ours in the timing of GMA, characteristics of the study populations, and definitions of respiratory exposure, which limits direct comparison.

Respiratory illness can involve intermittent hypoxia, inflammation, and cerebral haemodynamic instability, all of which may disrupt cerebral maturation [15,20,23]. Lee et al. found smaller cerebral white matter volumes and altered microstructure in several major white matter pathways among preterm infants with BPD but without focal abnormalities on conventional MRI [42]. Similarly, Grelli et al. showed that cumulative exposure to supplemental oxygen and airway pressure predicted white matter injury at term-equivalent age [43]. These observations indicate that the duration and intensity of respiratory exposure may reflect neurodevelopmental vulnerability that is not fully captured by a categorical BPD diagnosis. This distinction may help explain why the durations of respiratory support and mechanical ventilation differed across the fidgety movement groups, whereas clinically significant BPD did not.

Respiratory support, however, is closely related to GA, BW, and overall illness severity. Accordingly, the present findings do not show that respiratory exposure independently influences fidgety movement quality. The duration of respiratory treatment may instead reflect the combined effects of biological immaturity and neonatal clinical instability.

The most unexpected finding was the descriptive clinical profile of infants with abnormal fidgety movements. This group had the highest mean GA and BW and the shortest median durations of non-invasive respiratory support and mechanical ventilation among the three groups. Their values were therefore descriptively more favourable not only than those of infants with absent fidgety movements but also than those of infants with normal fidgety movements, although none of the abnormal–normal comparisons reached statistical significance after adjustment for multiple comparisons. To our knowledge, this specific pattern has not previously been reported using a three-category classification. Direct comparisons with previous studies are difficult because abnormal, sporadic, and absent fidgety movements have frequently been combined into a single aberrant or non-normal category [27,30].

One possible interpretation is that abnormal fidgety movements do not represent an intermediate level on an ordinal continuum from normal to absent fidgety movements. Domagalska-Szopa et al. identified different patterns of clinical risk factors associated with abnormal and absent fidgety movements, suggesting that these classifications may occur in infants with partly different clinical profiles [29]. The exaggerated amplitude, speed, or jerkiness that characterizes abnormal fidgety movements may therefore reflect altered regulation of motor activity that is not directly indexed by GA, BW, or respiratory-treatment duration. However, this interpretation remains speculative.

Second, the exclusion of infants with moderate or severe structural brain abnormalities and severe fetal growth restriction restricted the clinical risk range of the sample. Within this selected population, some less mature infants may retain normal fidgety movements, whereas abnormal fidgety movements may occur in relatively more mature infants in association with subtle microstructural, functional, prenatal, or environmental influences not detected by conventional MRI or the neonatal variables examined. Finally, the abnormal fidgety movement group was small (n = 11), making its estimates particularly susceptible to sampling variability. The observed profile should therefore be considered hypothesis-generating.

The cumulative neonatal morbidity count did not differ significantly across the fidgety movement groups. Similarly, the frequencies of clinically significant BPD, neonatal sepsis, NEC, and ROP did not vary significantly by fidgety movement classification. This finding is broadly consistent with Merino-Andrés et al., who found no association between a composite neonatal risk score and general movement quality during the writhing period [28], although differences in assessment timing and risk-score composition limit direct comparison.

The cumulative count used in the present study represented an unweighted sum of four clinically significant binary complications. Although the inclusion criteria ensured that the counted morbidities were clinically relevant, the score did not capture differences in their timing, duration, severity within the defined categories, or potential neurodevelopmental impact. Its low median values and restricted range may have further limited its ability to distinguish among the fidgety movement groups. Measures of the duration or intensity of neonatal exposure may therefore provide information that a simple count of diagnoses does not capture.

The distribution of normal and mildly abnormal term-equivalent MRI findings did not differ significantly across the fidgety movement groups. The sample size limited the precision of this comparison, and conventional categorical MRI assessment may not detect subtle microstructural or functional alterations. The absence of severe MRI abnormalities in the present cohort may also have reduced variability in both neurological risk and fidgety movement outcomes. Nevertheless, the presence of all three fidgety movement classifications within this selected cohort indicates that conventional term-equivalent MRI categories did not fully account for variation in fidgety movement quality. This finding should not be interpreted as evidence that the groups were neurologically equivalent, because subtle brain alterations were not assessed.

Clinically, GA, BW, and the respiratory course provide important information about overall neonatal and developmental risk. However, in the present sample, these characteristics did not reliably distinguish infants who subsequently showed normal fidgety movements from those with abnormal or absent fidgety movements. Although this study was not designed to develop or evaluate a predictive model, the findings suggest that birth characteristics and the apparent severity of the NICU course should not be used as proxies for later GMA findings. A relatively higher GA or BW or a less complicated respiratory course should not, by itself, be used to reassure families that fidgety movements will be normal or to determine that standardized functional assessment is unnecessary. Greater biological immaturity or longer respiratory treatment does not necessarily imply that fidgety movements will be abnormal or absent.

Current recommendations emphasize that early neurological risk assessment should integrate medical history with neuroimaging, standardized neurological examination, and standardized motor assessment, including GMA, rather than relying on any single source of information [7]. When counselling families, clinicians should explain that GA, BW, neonatal morbidity, MRI, and GMA provide related but non-interchangeable information. GMA contributes a direct functional assessment of the developing nervous system and should therefore be interpreted alongside, rather than replaced by, perinatal history and neuroimaging. Larger longitudinal studies are needed to determine whether combining these sources of information improves individualized developmental-risk prediction.

### Strengths and Limitations

A key strength of this study was its focus on preterm infants without moderate or severe structural brain abnormalities, as classified on term-equivalent MRI. This reduced the potential influence of major structural brain injury on fidgety movement classification. Analysing normal, abnormal, and absent fidgety movements separately also avoided combining potentially distinct movement patterns into a single non-normal category. In addition, the study examined both categorical neonatal morbidities and continuous measures of respiratory exposure.

The relatively small and unequal group sizes, particularly the small abnormal fidgety movement group, limited statistical precision. This was especially relevant for infrequent neonatal morbidities, and non-significant findings should not be interpreted as evidence of equivalence. Because several clinical indicators were examined, individual *p*-values should also be interpreted cautiously and alongside the reported effect sizes.

GA, BW, respiratory exposure, and overall illness severity are closely interrelated. Because each variable was analysed separately, their independent contributions to fidgety movement classification could not be determined. The limited sample size precluded reliable multivariable modelling, and the observed associations should not be interpreted as causal.

Although infants with moderate or severe structural brain abnormalities were excluded, conventional categorical MRI assessment may not detect subtle microstructural or functional alterations [44,45]. The study also lacked serial GMAs and long-term developmental follow-up and therefore could not examine changes in the general movement quality or determine the prognostic significance of the observed clinical differences. Finally, the single-centre setting and selected MRI profile may limit generalizability to the broader preterm population.

Future multicentre longitudinal studies should combine serial GMA, advanced neuroimaging, multivariable analyses, and long-term developmental follow-up.

## 5. Conclusions

Among preterm infants without moderate or severe structural brain abnormalities, GA, BW, and the durations of respiratory support and mechanical ventilation differed across fidgety movement classifications. However, significant pairwise differences were limited to infants with absent versus abnormal fidgety movements. Infants with absent fidgety movements had a lower GA and BW and required longer respiratory support and mechanical ventilation. Neither group differed significantly from infants with normal fidgety movements, and individual neonatal morbidities and the cumulative neonatal morbidity count did not differ across the three groups. These findings do not support a simple ordinal relationship between the examined clinical characteristics and normal, abnormal, and absent fidgety movements.

## Figures and Tables

**Table 1:** Demographic and clinical characteristics of the study sample.

Characteristic	Total Sample (n = 57)
Male sex, n (%)	33 (57.9)
Gestational age, weeks, median (IQR)	29.1 (27.4–32.0)
<28 weeks, n (%)	16 (28.1%)
28–<32 weeks, n (%)	24 (42.1%)
32–<37 weeks, n (%)	17 (29.8%)
Birth weight, g, mean ± SD	1389 ± 396
Caesarean delivery, n (%)	35 (61.4)
Bronchopulmonary dysplasia, n (%)	15 (26.3)
Mild	10 (17.5)
Moderate	5 (8.8)
Neonatal sepsis, n (%)	12 (21.1)
Necrotizing enterocolitis (Bell stage III), n (%)	4 (7.0)
Retinopathy of prematurity, n (%)	16 (28.1)
Stage 1–2	12 (21.1)
Treatment-requiring ROP	4 (7.0)
Non-invasive respiratory support, days, median (IQR)	24.9 (8.2–38.0)
Mechanical ventilation, days, median (IQR)	0.5 (0.0–5.0)
Clinically relevant neonatal morbidity count, median (IQR)	0 (0–1)

Note: IQR = interquartile range; SD = standard deviation. Continuous variables are presented as median (IQR) or mean ± SD, as appropriate.

**Table 2:** Term-equivalent MRI findings according to fidgety movement classification.

MRI Findings	Total Sample (n =57)	Normal FM (n = 24)	Abnormal FM (n = 11)	Absent FM (n = 22)
Corrected age at GMA, weeks	13.0 (12.1–14.3)	-	-	-
Normal MRI, n (%)	29 (50.9)	15 (62.5)	6 (54.5)	8 (36.4)
Mildly abnormal MRI, n (%)	28 (49.1)	9 (37.5)	5 (45.5)	14 (63.6)

Note: FM = fidgety movements; GMA = General Movements Assessment; MRI = magnetic resonance imaging. Data are presented as median (IQR) or n (%), as appropriate.

**Table 3:** Biological maturity and clinically relevant neonatal morbidities according to fidgety movement classification.

Variable	Normal Fidgety (n = 24)	Abnormal Fidgety (n = 11)	Absent Fidgety (n = 22)	Test Statistics	*p*	Effect Size
Gestational age, wks	29.90 ± 2.49	30.92 ± 2.57	28.32 ± 2.60	F (2,54) = 4.35	0.018	η^2^ = 0.139
Birth weight, g	1375 ± 302.7	1656 ± 502	1270 ± 381	F (2,54) = 3.87	0.027	η^2^ = 0.125
Moderate BPD, n (%)	2 (8.3)	0 (0.0)	3 (13.6)	χ^2^ = 1.714	0.505	V = 0.173
Neonatal sepsis, n (%)	5 (20.8)	1 (9.1)	6 (27.3)	χ^2^ = 1.460	0.535	V = 0.160
NEC Bell stage III, n (%)	1 (4.2)	1 (9.1)	2 (9.1)	χ^2^ = 1.315	0.595	V = 0.152
Treatment-requiring ROP, n (%)	1 (4.2)	0 (0.0)	3 (13.6)	χ^2^ = 2.606	0.403	V = 0.214
Non-invasive respiratory support, days	23.3 [7.4–36.9]	12.2 [3.5–22.4]	35.2 [23.6–54.0]	H (2) = 8.52	0.014	ε^2^ = 0.121
Mechanical ventilation, days	0.13 [0.0–3.5]	0.0 [0.0–0.8]	2.5 [0.5–9.9]	H (2) = 8.25	0.016	ε^2^ = 0.116
Clinically relevant neonatal morbidity count	1 [0–1]	0 [0–0.5]	1 [0–1]	H (2) = 2.25	0.324	ε^2^ = 0.005

Note: BPD = bronchopulmonary dysplasia; NEC = necrotizing enterocolitis; ROP = Retinopathy of prematurity. Data are presented as mean ± SD, median (IQR), or n (%), as appropriate. Overall group differences were examined using one-way ANOVA, Welch’s ANOVA, Kruskal–Wallis tests, or permutation-based categorical tests. Significant ANOVA results were followed by Tukey HSD tests. Significant Kruskal–Wallis results were followed by Dunn tests with Holm adjustment. Statistically significant *p*-values are shown in bold.

**Table 4:** Pairwise comparisons following significant one-way ANOVA.

Variable	Comparison	Mean Difference	T	Unadjusted *p*	Tukey-Adjusted *p*	Hedges’ g
Gestational age, wks	Normal vs. Abnormal	−1.02	−1.104	0.274	0.516	−0.398
	Normal vs. Absent	1.57	2.094	0.041	0.101	0.609
	Abnormal vs. Absent	2.60	2.763	0.008	0.021	0.979
Birth weight, g	Normal vs. Abnormal	−281.61	−2.050	0.045	0.110	−0.734
	Normal vs. Absent	104.48	0.938	0.352	0.619	0.300
	Abnormal vs. Absent	386.09	2.772	0.008	0.021	0.888

Note: Pairwise comparisons were performed using Tukey’s honestly significant difference test. Mean differences were calculated as the first group minus the second group. Pairwise effect sizes are presented as Hedges’ g.

**Table 5:** Pairwise comparisons following significant Kruskal–Wallis tests.

Variable	Comparison	Z	Unadjusted *p*	Holm-Adjusted *p*	r = Z/√N
Non-invasive respiratory support, days	Normal vs. Abnormal	1.35	0.178	0.178	0.228
	Normal vs. Absent	−1.87	0.062	0.124	0.275
	Abnormal vs. Absent	−2.819	0.004	0.014	0.491
Mechanical ventilation, days	Normal vs. Abnormal	1.21	0.228	0.228	0.160
	Normal vs. Absent	−1.94	0.053	0.106	0.256
	Abnormal vs. Absent	−2.74	0.006	0.019	0.363

Note: Pairwise comparisons were performed using Dunn’s test with Holm adjustment. Effect size was calculated as r = Z/√N, where N represents the combined sample size of the two compared groups.

## Data Availability

The data presented in this study are available from the corresponding author upon reasonable request.

## Abbreviations

The following abbreviations are used in this manuscript:

BPD	bronchopulmonary dysplasia
BW	birth weight
CA	corrected age
GA	gestational age
GMs	general movements
GMA	General Movements Assessment
MRI	magnetic resonance imaging
NEC	necrotizing enterocolitis
NICU	neonatal intensive care unit
ROP	retinopathy of prematurity
